# The multifaceted mechanisms of malignant glioblastoma progression and clinical implications

**DOI:** 10.1007/s10555-022-10051-5

**Published:** 2022-08-03

**Authors:** Rui Sun, Albert H. Kim

**Affiliations:** 1 Department of Neurological Surgery, Washington University School of Medicine, St. Louis, MO 63110 USA; 2 The Brain Tumor Center, Siteman Cancer Center, Washington University School of Medicine, St. Louis, MO 63110 USA

**Keywords:** Glioblastoma, Invasion, Extracellular matrix, Immune cell, Glioma stem cell, Tumor microenvironment

## Abstract

With the application of high throughput sequencing technologies at single-cell resolution, studies of the tumor microenvironment in glioblastoma, one of the most aggressive and invasive of all cancers, have revealed immense cellular and tissue heterogeneity. A unique extracellular scaffold system adapts to and supports progressive infiltration and migration of tumor cells, which is characterized by altered composition, effector delivery, and mechanical properties. The spatiotemporal interactions between malignant and immune cells generate an immunosuppressive microenvironment, contributing to the failure of effective anti-tumor immune attack. Among the heterogeneous tumor cell subpopulations of glioblastoma, glioma stem cells (GSCs), which exhibit tumorigenic properties and strong invasive capacity, are critical for tumor growth and are believed to contribute to therapeutic resistance and tumor recurrence. Here we discuss the role of extracellular matrix and immune cell populations, major components of the tumor ecosystem in glioblastoma, as well as signaling pathways that regulate GSC maintenance and invasion. We also highlight emerging advances in therapeutic targeting of these components.

## Introduction

**Fig. 1:**
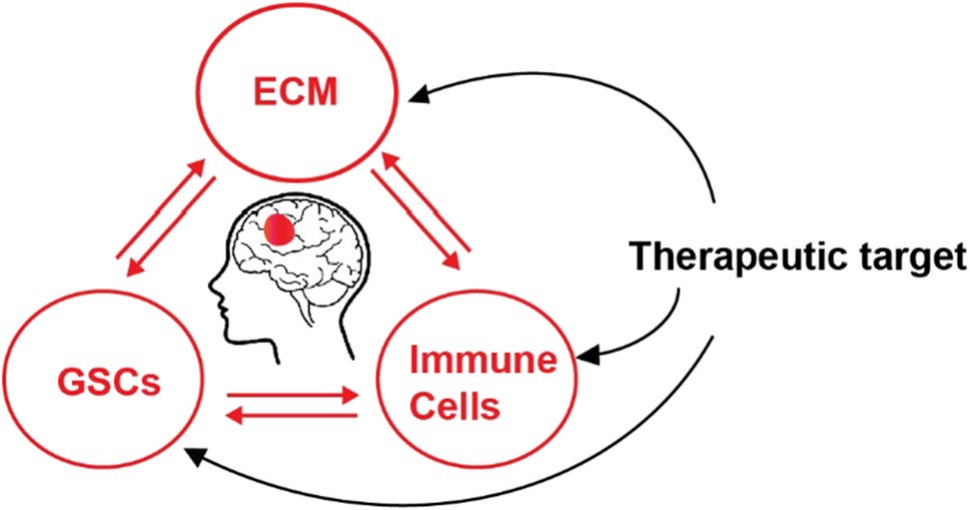
Potential therapeutic targets in the tumor ecosystem of GBM. ECM: extracellular matrix, GSC: glioma stem cell

Glioblastoma (GBM) is the most common malignant primary brain tumor in adults and is almost universally fatal. Recurrence is inevitable despite multimodal therapy, including surgery followed by adjuvant chemoradiation [1, 2]. There is an urgent need for novel and effective treatment strategies for GBM. In other cancers, immunotherapy, which harnesses the body’s immune system, has recently made historic breakthroughs through clinical successes via the use of immune checkpoint blockade (ICB) against both the CTLA-4 and PD-1 systems [3–6]. However, most clinical studies using ICB treatment demonstrated limited efficacy in improving the survival of GBM patients [7, 8]. The unique environment of the brain immune system may underlie the differential effects of immunotherapy on GBM compared to other types of tumor. Among the hallmarks of cancer, GBM exhibits extreme diffusion and invasion [9, 10]. Clinically, the median overall survival (OS) time for newly diagnosed GBM patients is 15 months due to the continuous infiltration of tumor cells despite maximal therapy [11]. Potentially, a more comprehensive understanding of the tumor microenvironmental landscape in GBM and brain-specific mechanisms of immunity may create new therapeutic opportunities for this challenging disease. The cellular constituents of a GBM clearly play a major role in the capacity of a tumor to progress and can predict prognosis. A GBM tumor contains a diversity of immune cells including local resident microglia, monocyte-derived macrophages (MDMs), monocytes, myeloid-derived suppressor cells (MDSCs), dendritic cells (DCs), neutrophils, and NK and T cells. We herein discuss various mechanisms involved in *IDH1/2*^wt^ GBM invasion and recurrence based on the composition of the tumor ecosystem as well as related potential druggable targets (Fig. 1).

## Extracellular matrix and GBM

**Fig. 2:**
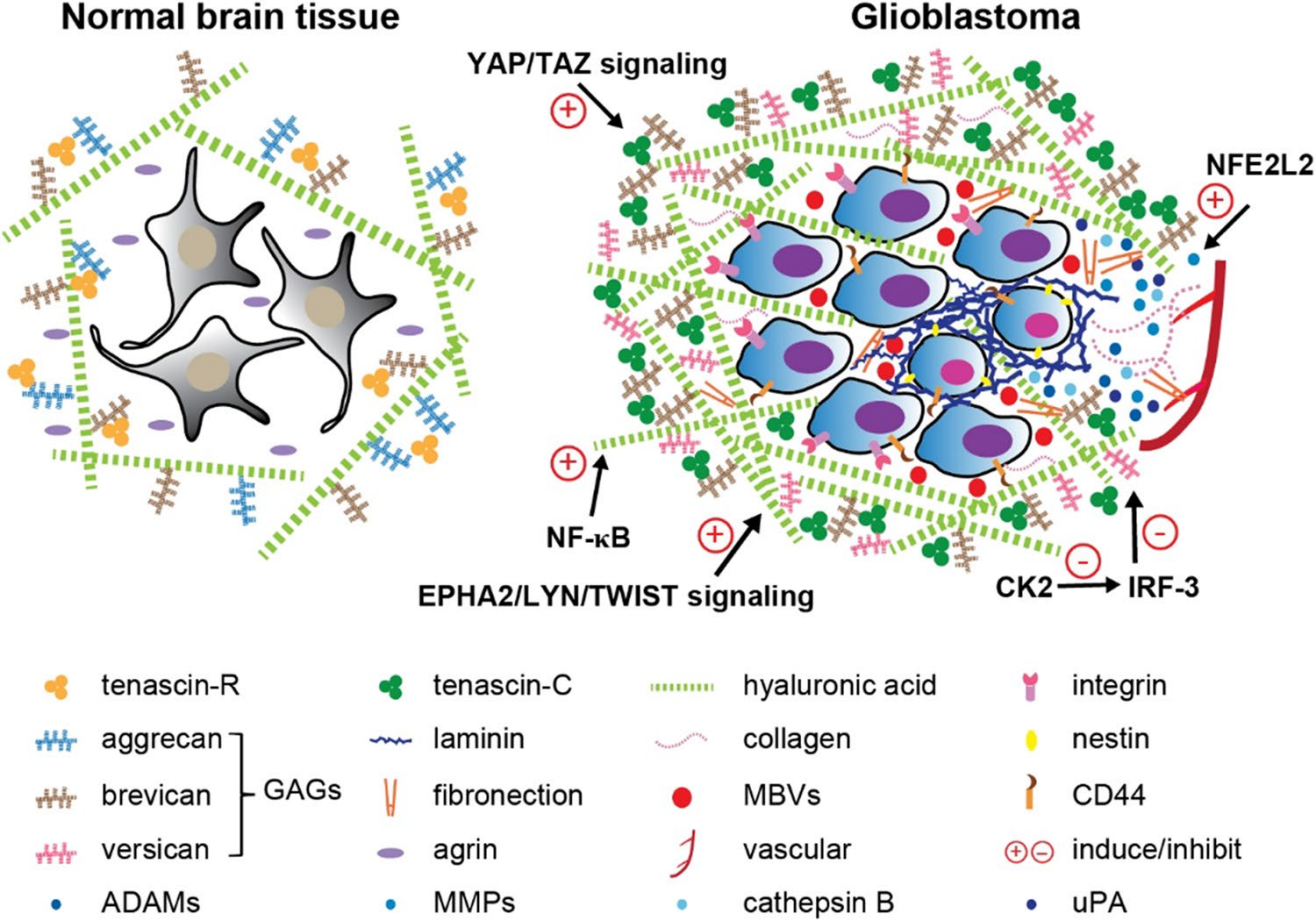
Reorganization of the extracellular matrix in GBM. Left: normal brain ECM components. Right: the changes in ECM composition and signaling pathways involved in increased structural stiffness, cell–cell information exchange, and tumor invasion

Progressive infiltration is a dynamic process to remodel the extracellular matrix (ECM), which is dominated by tumor cells with the utmost migratory potential [12]. The neural ECM is mainly deposited by glia and constitutes around 20% of the brain volume in adults [13]. In physiological states, brain ECM is distinguished by its richness in glycosaminoglycans, such as hyaluronan; proteoglycans, such as aggrecan, brevican, neurocan, phosphacan, and versican; and glycoproteins, such as link proteins and tenascins [14]. Moreover, brain vascular basement membranes form a three-dimensional (3D) protein network, which is predominantly composed of laminins, collagen IV isoforms, nidogens, and heparan sulfate proteoglycans [15]. Relatively few fibrillar proteins including fibronectin are found in brain vascular basement membranes, though they are abundant in other tissues [14]. The basement membrane plays two critical functions: (1) an important component of the blood–brain barrier (BBB) to protect from endogenous or exogenous dangerous signals and (2) spatially separating endothelial cells from neurons and glial cells [16]. Tumor cells can evolve their ability to co-opt certain components of the ECM to assist in cell survival and invasion [16]. In addition, high-grade malignant cancers are frequently associated with abnormal vascularization, which is believed to facilitate the motility and invasiveness of tumor cells [17]. We review recent discoveries that focus on tumor invasion through the lens of dynamic alterations of ECM components in GBM tumors (Fig. 2).

Recurrent GBMs often exhibit a mesenchymal, stem-like phenotype with a stiffened ECM to potentiate their proliferative and invasive ability [18–20]. High levels of glycoproteins including tenascin-C are secreted by tumor cells and increase the bulkiness of the glycocalyx of ECM [18]. A bulky glycocalyx reciprocally interacts with integrins of tumor cells and strengthens tissue tension [18, 19]. Depending on the ECM-integrin signaling, infiltrative tumor cells are active and motile [19, 20]. Thus, blocking the increase in glycoproteins and glycocalyx bulkiness could disrupt the adaptive circuit between tumor cells and ECM, which may represent a therapeutic target for GBM. The content and structure of heparan sulfate glycosaminoglycans (GAGs) are associated with tumor cell adhesion and invasion in both human and mouse GBM tumorspheres *in vitro* [21]. One study, using both rat F98 GBM cells and human patient-derived glioma stem cells, demonstrated that the invasive spread of GBM is correlated with increased expression of chondroitin sulfate proteoglycan (CSPG)-associated GAGs in ECM and showed proof-of-concept that an inhibitor of sulfated GAG signaling can decrease tumor dissemination *in vivo* [22]. In brain tumors, expression levels of proteoglycans are highly regulated, such as CSPG4/NG2, PTPRZ1, CD44, aggrecan, brevican, and versican [23]. Among the membrane-associated proteoglycans, the most upregulated, including CSPG4/NG2, PTPRZ1, and CD44, function to maintain self-renewal, suppress differentiation, and assist in the migration of GBM cells, contributing to tumor invasion and recurrence [24]. Very few aggrecans exist in the GBM ECM [25, 26]. The production of brevican and versican is greatly increased in GBM cells, which increases cell motility and promotes late-stage tumor migration [27–30]. GBM migration can be influenced by the molecular weight of hyaluronic acid (HA) [31]. In response to radiation, HA production is strongly induced through NF-κB signaling and contributes to the invasiveness of GBM cells by providing a migration tract in the ECM [31, 32]. Two migration modes have been identified in cancers: mesenchymal and amoeboid [33, 34], and mesenchymal migration is the major mode that has been found in GBM [35]. Recently, HA was discovered to induce Rho-associated protein kinase (ROCK)-dependent amoeboid migration in GBM cells, which has been suggested to be a salvage mechanism adopted by tumor cells and associated with therapeutic resistance [36–38]. In mammals, laminins are a large family of trimeric basement membrane proteins which have also been identified as critical constituents of the stem cell niche [39, 40]. Several investigations have found aberrantly high levels of laminins in extremely aggressive GBMs, a phenomenon associated with recurrence and mortality [41–43]. Of note, the overexpression of laminin-411, one major isoform of vascular laminins, correlates with the expression of cancer stem cell (CSC) markers in tumor tissues, such as CD133, Nestin, c-Myc, and Notch pathway members as well as shorter survival of GBM patients [43]. Depletion of laminin-411 in GBM cells using CRISPR/Cas9 reduced tumor progression and increased the survival of tumor-bearing animals [43]. In contrast, the content of agrin, a heparan sulfate proteoglycan that contributes to the basal lamina of the BBB capillary, is significantly reduced in human GBM, leading to the breakdown of existing BBB and accelerated tumor migration [44–46]. The synthesis of agrin was found to be negatively correlated with the expression of tenascin-C, accumulation of which activates angiogenesis [44]. In addition, fibronectin (FN), which is typically associated with the vascular wall in the body but less abundant in normal brain, was found to be robustly induced in GBM [47, 48]. FN functions as a fibrillar scaffold to support the assembly of ECM with other proteins and a cell–matrix/cell–cell communication hub, thereby facilitating signal transduction [49–51]. FN reduction by short-hairpin RNA (shRNA) significantly inhibited tumor cell adhesion and motility [48]. Finally, diverse collagens were found to be overproduced in the ECM of mesenchymal GBM tumors and drive tumor cell invasion [52]. Collagens were observed at tumor boundaries in focal mouse GBM xenografts but in invasive xenografts, were found to be interspersed with tumor cells to form a complex [53]. Additional 3D ECM/tumor cell *in vitro* systems suggest collagen is indispensable for matrix preparations in assays of tumor cell behavior, providing circumstantial but functional evidence for a role in tumor growth and invasion [54, 55]. In summary, invasive GBM cells remodel the tumor microenvironment by upregulating or downregulating the synthesis of ECM components and vascular basement membrane proteins to generate a more hospitable environment for tumor migration and infiltration (Fig. 2).

Most ECM elements are tightly regulated in the TME, and a dynamic and coordinated process creates a pro-survival and migratory microenvironment for GBM cells. Therefore, for ECM-targeted therapeutics, suppressing the kinetic transformation of the ECM composition would be more effective than merely targeting the synthesis of certain components to slow tumor progression. Several proteases such as uPA (a serine protease), cathepsin B (a cysteine protease), MMPs (zinc-dependent matrix metalloproteases), and ADAMs (transmembrane and secreted proteases), which regulate ECM degradation, have been suggested to be druggable candidates [56]. Moreover, determination of the initial signaling events in GBM TME that activate ECM remodeling may aid in developing efficacious treatments to prevent tumor recurrence and invasion. For instance, IRF3-mediated transcriptional events have been shown to repress the expression of pro-invasive ECM genes in GBM. Therefore, the inhibition of casein kinase 2 (CK2), a negative regulator of IRF3, might potentially restrict GBM invasion [52]. NFE2L2, an important regulator of oxidative stress, is upregulated in chemotherapy-resistant GBM cells. NFE2L2 can increase MMP2 expression, which is an ECM remodeling marker and mediates tumor chemo-resistance [57]. Targeting NFE2L2 may represent an alternative strategy to increase efficacy of temozolomide (TMZ) and decrease the odds of tumor recurrence [57].

A recent discovery revealed a newly appreciated constituent in ECM, matrix-bound nanovesicles (MBVs) [58]. Like exosomes, MBVs belong to the extracellular vesicle family, which can house multiple biological effectors, including enzymes, lipids, growth factors, cytokines, chemokines, DNA fragments, and microRNAs (miRNAs) [58]. Multifaceted roles of ECM MBVs in promoting an oncogenic environment in GBM have been reported, such as inducing angiogenesis, reprogramming metabolic pathways, immunomodulation of the TME, polarizing immune cells to an immunosuppressive mode, and promoting tumor cell invasion [59]. As a courier, MBV mediates the information exchange between different types of cells by delivering ligands to receptors to alter the phenotypes and functions of recipient cells [60]. MBVs may thus play important roles in drug resistance and GBM relapse. On one hand, interrupting the machinery of information delivery by MBVs may be an effective strategy to restrain GBM infiltration and regrowth. On the other hand, anti-tumor drugs including small molecule inhibitors and monoclonal antibodies can be packaged into MVBs to be delivered into tumor sites due to their unique ability to cross the BBB [61]. Moreover, based on their content and activities, MBVs can be used as biomarkers for diagnosis and treatment responses [62]. Hence, thoroughly understanding the mechanisms of how MVBs are secreted and function may provide insights into GBM progression and new treatment options.

In recent years, accumulating studies have turned their attention to the mechanical properties of ECM in the TME [63–73]. Matrix stiffness is an intrinsic mechanical property of ECM and believed to be influential in tumor progression and clinical outcome [64, 65]. Tumor cell proliferation, migration, and invasion are always accompanied by alterations in the density and constitution of ECM which modulate matrix stiffness and mechanosignaling in the TME [66, 67]. Distinct from soft connective tissues under homeostasis, tumor ECM remodeling usually leads to increased stiffness [68]. Several brain ECM components suggested to be the main contributors of ECM stiffness are overexpressed during GBM progression, such as tenascin-C, HA, brevican, and fibronectin [69, 70]. Large amounts of increased ECM components generate physical stress and mechanical cues in the TME. The following mechanotransduction activates a series of biochemical pathways, including cell mitosis, cytoskeleton contraction, and cell motility [68]. For instance, increased HA production induces a more stiffened ECM which produces mechanical stress to drive F-actin expression and cellular morphological changes in GSCs, thereby boosting tumor cell adhesion and movement [71]. Furthermore, GBM tumors are characterized by hypercellularity and a very high rate of mitosis [68]. Rapid proliferation leads to higher cell density inside the tumor than neighboring regions, causing enhanced solid and interstitial pressure that further drives tumor cells infiltration into surrounding areas [72]. Inoculation of tumor cells into fibronectin-coated two-dimensional (2D) ECM with defined mechanical rigidity showed that the diffusive potential of GBM cells correlated with the degree of ECM stiffness [73]. To recapitulate the 3D tumor niche, bioengineered 3D brain tumor models have been developed to help elucidate the effects of matrix stiffness on GBM cell behavior using polyethylene glycol (PEG)-based hydrogels which mimic the biochemical and mechanical properties of brain [74]. Low hydrogel stiffness enhanced patient-derived glioblastoma xenograft GBM cell proliferation, and high stiffness supported robust GBM cell spreading in the tumor niche [75]. A positive-feedback signaling loop was speculated to exist between GBM invasiveness and ECM rigidity, which could contribute to progressive tumor cell infiltration in brain parenchyma. Altogether, it appears that during GBM development, cell proliferation, progressive infiltration, and sustained ECM stiffening occur simultaneously and are spatially entangled. Overall, based on the physical characteristics of ECM stiffness, tumor phenotype and treatment may vary [68]. A full understanding of mechanisms regulating the dynamic interplay between ECM and GBM cells is needed and may provide innovative therapeutic approaches for GBM.

Several investigations have identified important signaling pathways that respond to ECM mechanical cues [76–83]. One group discovered in breast cancer that increased ECM rigidity could activate the EPHA2/LYN/TWIST signaling pathway that promotes the epithelial-to-mesenchymal transition (EMT), and tumor metastasis [76]. Another group, based on a computational model, systemically analyzed the mechanisms of YAP/TAZ signaling nexus in integrating various mechanosensors and biochemical signals [77, 78]. Integrins are a class of transmembrane receptors and found transmitting critical mechanosignaling in the TME [79]. By binding ECM ligands, integrins drive multiple cell events including cytoskeleton reorganization and EMT, which mediate tumor initiation and migration [79]. Cellular metabolic processes, such as glycolysis and amino acid consumption in the TME, were also found involved in transformations between ECM biophysical properties and tumor aggressiveness [80–82]. Importantly, several mechanosensing signaling pathways have been demonstrated to be involved in the adaptive adjustment of the GBM TME, such as Hippo/YAP/TAZ, CD44, and actin skeleton signaling, which remodel the cytoskeleton and initiate biological processes including cell–cell/ECM interactions, proliferation, and migration/invasion of GBM cells, [83]. Since tissue stiffness is critical in driving cancer progression, mechano-targeted therapy has been tested clinically [84]. For example, Shen and colleagues used renin-angiotensin inhibitors that target myofibroblast-mediated tissue stiffness, in the treatment of metastatic colorectal cancer [85]. Patients who received anti-angiogenic therapy showed improved survival when concomitantly treated with renin-angiotensin inhibitors [85]. Although targeting tissue stiffness and other mechanical properties for cancer therapy looks promising, signaling complexities in the TME make present therapeutic interventions less effective [84, 85]. Hence, a comprehensive understanding of the signaling network associated with different ECM components and mechanical forces would be extremely important for improving GBM treatments in future.

## Leukocytes and GBM

GBM is characterized by profound cellular heterogeneity in both tumor cells and tumor-associated leukocytes, which has led to overall less robust clinical responses to immunotherapy compared to metastatic brain tumors [86]. Moreover, these diverse cellular components can contribute to tumor progression and prognosis. GBM shows poor response to immunotherapies compared to more immunogenic cancers that have a less immunosuppressive TME [87]. Due to the low immunogenicity of GBMs, which generally harbor fewer neoantigens and neoepitopes, most tumor cells escape from immune surveillance and attack. Though there are abundant immune cells in GBM TME, they are not fully activated by antigens but transform into immunosuppressive cells, thereby contributing to a pro-tumor local environment. It has been reported that immune cell populations enriched in GBM TME include local resident microglia, monocyte-derived macrophages (MDMs), dendritic cells (DCs), neutrophils, monocytes, myeloid-derived suppressor cells (MDSCs), NK (natural killer), and T cells [86–88]. Herein, we discuss the role of immune cells in regulating GBM growth and invasiveness. We also describe promising therapeutic strategies that can be used to improve anti-GBM immunotherapy.

### Tumor-associated Macrophages

**Fig. 3:**
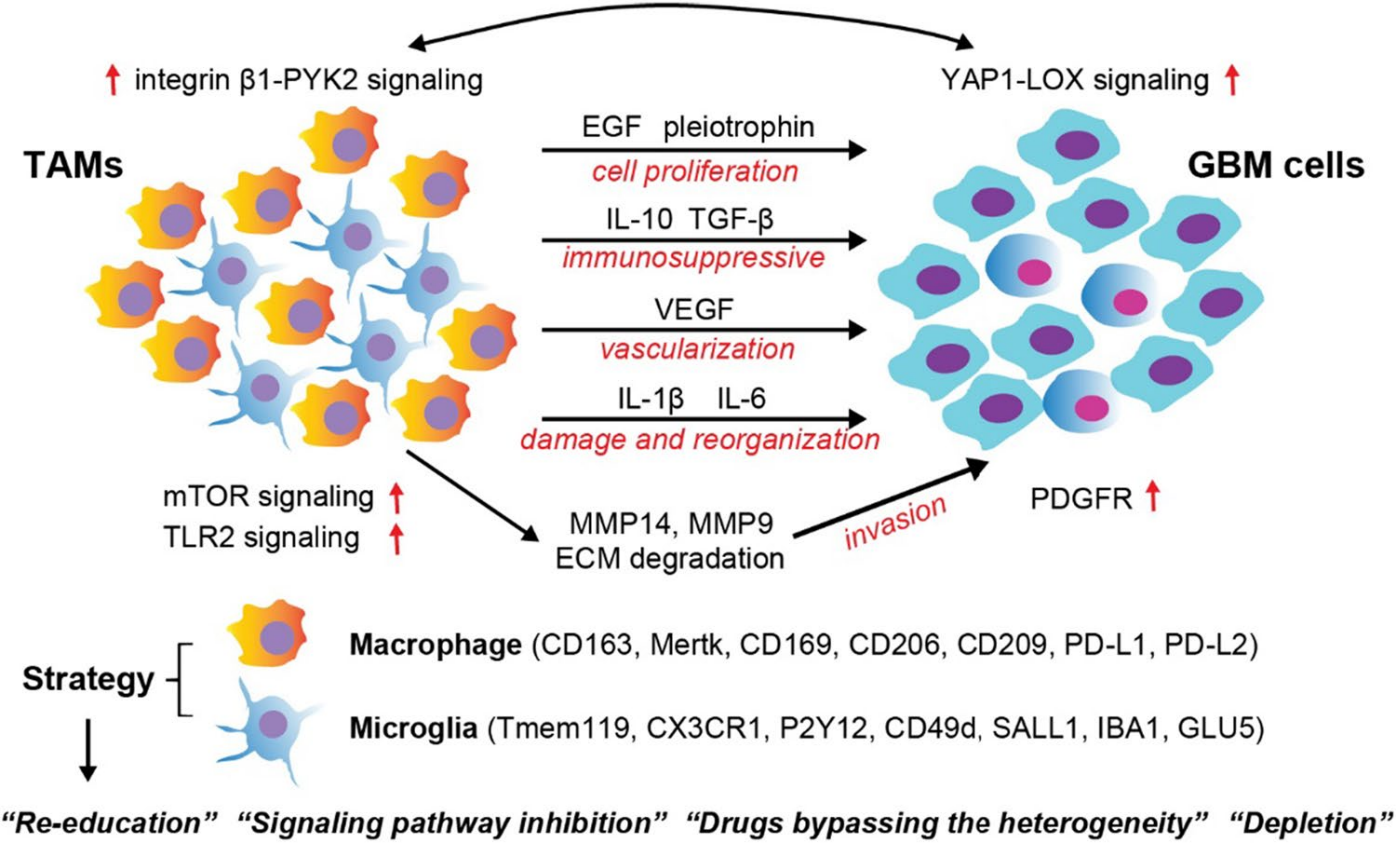
TAMs contribute to tumor growth and infiltration. By secreting cytokines and growth factors: TGF-β, IL-10, IL-6, IL-1β, pleiotrophin, EGF, and VEGF, TAMs can promote tumor cell proliferation, angiogenesis, ECM reorganization, and immunosuppression. By secreting enzymes (MMPs), TAMs can assist tumor cell migration and invasion. Approaches used for TAM-targeted therapeutics include re-education of phenotypes, signaling pathway inhibition, drugs bypassing the heterogeneity, and cell subpopulation depletion

Among immune cell populations in GBM TME, tumor-associated macrophages (TAMs) are predominant [89], comprising up to 50 ~ 70% of leukocytes [90]. Phenotypes and functions of TAMs are highly plastic depending on the TME [91]. TAMs encompass two major populations: tissue-resident microglia, and recruited monocyte-derived macrophages (MDMs) [91] (Fig. 3). Microglia as brain-resident macrophages are distinct from other terminal-differentiated and hematopoietic-derived macrophages, constituting the main part of the brain neuroimmune system [92]. Equipped with the sensome, such as various pattern-recognition receptors (PRRs), chemoattractant and chemokine receptors, purinergic receptors, receptors for ECM proteins, and cytokine receptors, microglia can react to potential harmful stimuli by going through a series of morphological and functional changes [91, 93]. Infiltrative tumor progression promotes the recruitment of blood-borne monocytes and MDM expansion in the brain TME [86]. There are more accumulated MDMs in GBM than in *IDH1/2*^mut^ grade 4 astrocytoma and low-grade gliomas [86]. An established concept is that TAM enrichment is generally associated with poor prognosis [94]. To date, considerable studies have been performed using experimental mouse models of GBM or limited samples from GBM patients to define the role of TAMs in GBM progression from the cytogenetical or immunological angles [89–91]. The plasticity of TAMs endows them with flexible features to adapt to the local inflammatory status and normally allows the elimination of infectious agents without undue tissue damage. This intrinsic plasticity allows TAMs to be reprogrammed by the local brain TME and change into distinguishable differentiation trajectories [95]. The dichotomy of TAMS into an M1 or M2 phenotype generally parallels anti- or pro-tumorigenic functions in GBM. TAMs are known to contribute to tumor growth, infiltration, and neovascularization via multiple mechanisms, such as secretion of cytokines and growth factors—TGF-β, IL10, IL6, IL1β, pleiotrophin, and EGF [96]. More recently, by utilizing high-depth single-cell RNA sequencing, a recent study observed that myeloid cells, especially macrophages, which are widely distributed both in the tumor core and the surrounding peritumoral space, differentially express inflammation-related genes [97]. Tumor-infiltrating macrophages and brain-resident microglia preferentially occupy the tumor and peritumoral spaces, respectively [97, 98]. Moreover, TAMs express inflammation-related genes with spatial specificity: in the tumor core, TAMs highly express immunosuppressive and pro-angiogenic genes, such as *IL1RA*, *TGF-β1*, *HIF1A*, and *VEGFA*, whereas proinflammatory markers *IL1A* and *IL1B* are significantly upregulated in TAMs located in the tumor periphery [97]. However, peripheral TAMs are found to continually evolve towards the anti-inflammatory state [98]. Furthermore, infiltrating neoplastic cells are scattered in the tumor peripheral region, with increased expression of migration and invasion-related genes, including *ECM2*, *ANGPT1*, and *TSPAN7* [97]. A signaling loop YAP1-LOX-integrin β1-PYK2 was found to mediate a symbiotic interaction between recruited macrophages and GBM tumor cells [99]. The YAP1-LOX pathway was abnormally activated in *PTEN*-deficient GBM cells, which caused macrophage chemoattraction via activation of the integrin β1-PYK2 pathway in macrophages. Increased macrophage infiltration promoted GBM cell survival and migration [99]. Macrophages can also drive GBM cells into the mesenchymal-like (MES-like) state, which might influence the response to immunotherapies [100]. The platelet-derived growth factor (PDGF) signaling pathway plays an important role in driving GBM progression. Enhanced PDGF receptor (PDGFR) expression correlated with poor prognosis in GBM patients [101]. M2-polarized microglial cells, rather than MDMs, were found to induce PDGFR overexpression in a subset of GBM cells. Increased PDGFR signaling potentiated the migratory and invasive capacity of tumor cells [102]. GL261 glioma-derived soluble factors can activate Toll-like receptor 2 (TLR2), which is highly expressed in tumor-associated microglia [103]. TLR2 signaling upregulated microglia-derived MMP14 and MMP9 production, which accelerate ECM degradation and reorganization and induce tumor cells to move and invade [103, 104]. Additionally, it has been reported that microglia could be re-trained by GBM-initiating cells by inducing mTOR signaling in microglia but not MDMs in both *in vitro* and *in vivo* GBM mouse models [105]. The mTOR-dependent regulation of STAT3 and NF-κB activity promoted an immunosuppressive microglial phenotype, thereby contributing to tumor immune evasion and invasive growth *in vivo* [105]. Microglia can also be reprogrammed by radio- and/or chemotherapy to create new stress-responsive states, which can support GSC migration and contribute to tumor recurrence [106, 107]. In short, the crosstalk between TAMs and tumor cells remodels the functional state of TAMs to accelerate tumor progression and mediate resistance against current therapeutics (Fig. 3).

Transcriptional programs and extensive immune marker analysis from large cohorts of patients revealed that compared to *IDH1*^mut^ gliomas, which are dominated by TAMs of microglial origin, *IDH1*^wt^ GBMs show an obvious increase of MDM TAM invasion in the TME. The biological process of monocyte/MDM differentiation in the TME is associated with the downregulation of monocyte makers, including CCR2 and CD33, and increased expression of macrophage markers, such as CD163 and Mertk [86]. However, according to expression levels of other markers including CD169, CD206, CD209, PD-L1, and PD-L2, MDM TAMs can be further divided into different subsets [86]. At resting state, brain-resident microglia can be distinguished from MDMs based on the expression of several immune cell makers, such as CD49d, CX3CR1, Tmem119, and P2Y12 [86, 107]. To identify reactive microglia from TAMs, other molecules have been used, such as SALL1, a transcriptional regulator [108–111]. Though the specific role of GLUT5, a member of transmembrane transporters for fructose, remains still uncertain in microglia, several studies suggested it could be a useful marker for both resting and activated microglia [111, 112]. Overall, phenotypes of TAMs in GBM TME are multiple and variable (Fig. 3). Though in-depth investigations of the immune landscape of GBM may be valuable in identifying heterogenous TAM populations, the complexity and flexibility of TAM phenotypes make it difficult to develop effective and durable TAMs-targeted immunotherapy.

For now, several strategies can be envisioned to therapeutically target TAMs: (1) re-educate TAMs into cell populations with common anti-tumor characteristics; (2) target major determinants that convert MDM lineages into tumor-promoting cells; (3) restrict the heterogeneity of TAMs; (4) block MDM TAM induction or deplete all TAMs (Fig. 3). Macrophage colony-stimulating factor (M-CSF/CSF1) signaling through its receptor (CSF1R) is vital for the differentiation of myeloid progenitors into the monocyte/macrophage cell lineage [113]. CSF1 also exerts diverse effects on macrophage functions, such as survival, proliferation, migration, and maintenance [114, 115]. Anti-CSF1/CSF1R treatment in GBM initially seemed feasible as a therapeutic option to restrict TAM development and survival. The first CSF1R blockade experiment in a mouse proneural GBM model showed a survival advantage in tumor-bearing mice and reduced tumor growth and invasion [116]. However, in this study, TAMs were not depleted completely due to tumor cell-derived granulocyte–macrophage CSF (GM-CSF) and interferon γ (IFN-γ) which facilitated TAM survival [116]. A following study revealed long-term CSF1R inhibition led to obvious drug resistance and inability to promote sustained animal survival [117]. The suggested causes for this failure included dormant tumor cells, remaining TAMs, and TME-driven crosstalk between TAMs and tumor cells [117]. Consistently, a clinical trial (NCT01349036) failed to show efficacy of an oral CSF1R inhibitor in recurrent GBM [118]. Thus, although a TAM depletion strategy showed much more promise in other tumor types [119–121], targeting TAMs in GBM seems more complicated and challenging. More recently, CSF1R inhibitor combined with radiotherapy substantially enhanced animal survival in preclinical GBM models, suggesting that CSF1R inhibition might mitigate myeloid mechanisms of radiation resistance [122]. In addition, since it is well-known that the CCL2/CCR2 axis is essential for monocyte migration into the inflamed CNS [123], another way to decrease TAM accumulation in GBMs might be to target the CCL2-CCR2 axis [90]. It has been speculated that CCR2-dependent myeloid cells also play crucial roles in controlling glioma growth. Recently, one study reported that combining CCR2 blockade with anti-PD-1 treatment could extend survival in KR158 glioma-bearing mice [124, 125]. Furthermore, reprogramming TAMs into cells with an anti-tumor phenotype has been tested *in vivo*. Inducing M1-like TAMs by IL-12 contributed to increased efficacy of a triple immunotherapy in the 005 GBM model [126]. Taken together, these recent findings offer a glimmer of hope about TAMs-targeted therapy and highlight the importance of combination therapy. Given that TAMs are adaptive and respond to cues from tumor cells, further investigation of how GBM cells hijack TME components and exploit TAMs to serve tumor growth and immune evasion may yield new treatment strategies.

### Dendritic cells

Dendritic cells (DCs) are myeloid-derived, specialized antigen-presenting cells (APCs) that initiate adaptive immune responses and function as the “sentinels” of the immune system. DCs are highly potent in capturing antigens to form peptide-major histocompatibility complex (MHC) molecule complexes that can be recognized by T cell receptor (TCR), and thus the key to activate naive T cells [127–129]. There are two major subsets of DCs: myeloid DCs (mDCs; also known as conventional DCs or classical DCs) and plasmacytoid DCs (pDCs) [130, 131]. mDCs are more equipped for the generation of CD8^+^ T cell-mediated immune responses, while pDC subset is often involved in a rapid anti-virus immunity [132]. Hence, DCs undertake the pivotal role in bridging tumor recognition and T cell-mediated tumor elimination. DCs have been actively studied as a target in efforts to generate efficacious combination therapies in various clinical trials [133, 134]. However, in the central neuroimmune system, DCs have been believed to play a lesser role compared to microglia and MDMs [135, 136]. Of note, in GBM, very few DCs exist and are much less abundant than TAMs/monocytes, which comprise up to around 80% of total leukocytes with T cells occupying approximately 15% [86]. Thus, few reports have indicated how DC subsets affect GBM progression and their role in GBM malignancy has not been thoroughly explored. One investigation may provide some indirect clues and found that macrophage migration inhibitory factor (MIF) is highly expressed in GBM tumors and enhances tumor cell autophagy and migration by activating ROCK1 [137]. Further analysis indicated that MIF not only can block both immature and mature DCs migration but also suppresses DC maturation and function, which help GBM cells escape from immune surveillance [137].

Research on DCs have focused on their “adjuvant effect” to boost tumor immunogenicity in combination therapies for tumors. DC vaccines have been tested in several clinical trials for GBM therapy [138–145]. A preliminary study (BB-IND 12,903 and BB-IND 11,162) that vaccinated a small cohort of GBM patients with autologous tumor lysate-loaded DCs demonstrated that DC vaccination in combination with radiation and chemotherapy is feasible, safe, and may improve survival in a subset of patients [138]. Another study performed a clinical trial (EY-DOH-MD #0,910,072,504) that injected autologous DC-tumor vaccine in GBM patients that had undergone surgery and radiation treatment concluded that autologous DC-tumor immunotherapy can improve patient survival but may cause elevation in serum AST/ALT level [139]. Follow-up work (EudraCT 2006–002,881-20) suggested that full integration of autologous DC-based tumor vaccination into standard postoperative radiotherapy and chemotherapy for newly diagnosed GBM is safe and possibly beneficial [140]. One clinical trial (16–184-4412) created DC-glioma fusion cells and evaluated the efficacy of this fused-cell vaccine in recurrent GBM patients resistant to TMZ treatment [141]. Likewise, fused-cell immunotherapy was well-tolerated and safely induced anti-tumor effects in GBM patients [141]. However, recent clinical trials have showed contradictory results about the efficacy of DC vaccines in improving patient survival [142–145]. The phase II “GBM-Vax” trial (NCT01213407) showed no clinical efficacy in prolonging patient survival [142]. However, two studies demonstrated that DC vaccines were safe and increased progression free survival (PFS) in GBM patients [143, 145]. Intriguingly, the interim analysis from a large Phase III trial of an autologous dendritic cell vaccine in newly diagnosed glioblastoma (DCVax-L) showed promise in terms of overall survival and had a substantial number of long-term survivors, although the patient characteristics per treatment arm were not clearly described [146]. Thus, DC vaccines appear to have therapeutic potential but may require further optimization.

### Monocytes

As precursors of both macrophage and DCs, most monocytes circulate in the bloodstream before settling into tissues. Circulating monocytes show morphological, antigenic, and functional heterogeneity [147]. Three major populations have been defined phenotypically in humans: “resident” monocytes, which constantly replenish tissue-resident populations of macrophages and DCs at steady state, with a CD14^+^ CD16^+^ CD64^−^ CX3CR1^hi^ phenotype; “inflammatory” monocytes, the foremost functional population, which respond to inflammatory cues and migrate to the inflammation niche, and are identified by a CD14^hi^ CD16^−^ CD64^+^ CCR2^+^ CX3CR1^low^ phenotype; and “intermediate” monocytes, a group of pre-inflammatory population, which is also recruited by inflammatory stimuli and can differentiate into DCs, and are identified as CD14^+^ CD16^+^ CD64^+^ monocytes [147]. Most studies are drawn to the CCR2^+^ cell subset. CCR2^+^ monocytes tend to be recruited into inflamed tissues and serve resemble functions as macrophages and DCs [148]. In GBM TME, CCR2^+^ monocytes can have different differentiation trajectories and transform into heterogeneous macrophages accompanied by significant CCR2 downregulation [86]. Although lots of studies tend to functionally group TAMs and monocytes together, it has been reported that in *IDH1*^wt^ glioma cohorts, the frequency of monocyte subset correlated with patient outcome [86]. Thus, although monocytes are present at low frequencies in GBM TME, the functional role of monocytes during tumor progression needs to be more clearly defined.

One investigation showed in a mouse model of GBM that genetic loss of *cx3cr1* did not affect accumulation of TAMs in peritumoral areas, but instead resulted in high amounts of CX3CR1^low^ inflammatory monocytes localized to the perivascular area in the CNS [149]. CX3CR1^low/−^ monocytes overexpress IL-1β and high IL-1β level upregulates ICAM-1 and VCAM-1 expression in GBM cells, which support the GSC phenotype and accelerate tumor progression [149, 150]. It was found that accumulating monocytes in the TME can be re-educated by GBM cells and develop immunosuppressive features, thereby contributing to tumor growth [151]. Additionally, monocytes were found to be more resistant to radiation and chemotherapy, whereas the numbers of both effector CD4^+^ T and CD56^+^ NK cells were significantly decreased following the treatment [152]. It has been suggested that the cell subset of therapy-resistant monocytes contributes to generate an immunosuppressive TME after traditional therapy, which implies tolerant monocytes are closely related to tumor recurrence [152]. Although this may seem to indicate monocytes are associated with GBM progression and recurrence, the cues in the TME that dictate monocyte fate and differentiation into pro- or anti-tumor cells remain incompletely understood. With a better understanding of these molecular cues, targeting monocyte trafficking or differentiation in GBMs may represent an interesting therapeutic strategy in the future.

### Myeloid-derived suppressor cells

MDSCs are also myeloid-lineage and a heterogeneous group of immune cells, which are well-known for their strong immunosuppressive ability. During chronic infection and cancer, MDSCs expand and are frequently associated with poor prognosis and treatment resistance in patients [153]. Work over recent years is beginning to illuminate the role of MDSC in GBM progression, which overall indicate that MDSCs are functionally immunosuppressive. MDSCs generally consist of two groups of cells: granulocytic or polymorphonuclear (PMN-MDSCs) and monocytic (M-MDSCs). PMN-MDSCs are phenotypically and morphologically similar to neutrophils, whereas M-MDSCs resemble monocytes [154]. MDSCs are more immunosuppressive in tumors than in infectious diseases, and M-MDSCs are more prominent than PMN-MDSCs [155]. In addition, M-MDSCs have the potential to rapidly differentiate into TAMs [155]. Herein, we review recent progress about the role of MDSCs in regulating GBM progression and recurrence as well as possible clinical benefits of targeting this immune cell subpopulation.

MDSC number was found to be significantly elevated in the peripheral blood of GBM patients compared to that of brain tumor patients [156, 157]. In GBM patients, increased MDSC abundance is frequently predictive of tumor recurrence and poor clinical outcome [156]. MDSCs can highly express inhibitory molecules like PD-L1, arginase, reactive oxygen species (ROS), and IDO, and support regulatory T cell (Treg) induction that deters effector T cell proliferation and function [158]. MDSCs can also inhibit CD4^+^ effector-memory T cell (TEM) function in GBM by upregulating PD-1 expression in the T cell population which leads to T cell exhaustion [159]. MDSCs suppress cytotoxic activities of NK and NK T cells in the TME [160]. MDSCs were found in proximity to self-renewing GSCs in the brains of GBM patients, and the information exchange between MDSCs and GSCs facilitates tumor invasion [161]. GSCs secrete MIF that induces arginase-1 production in MDSCs, which represses cytotoxic T cell response and promotes GSC survival and migration [161]. M-MDSCs are found to express high levels of MIF cognate receptor CD74, whereas PMN-MDSCs highly express MIF non-cognate receptor CXCR2 [162]. It has now been established that males have a higher risk of GBM and exhibit more aggressive disease compared to females, suggesting sexually dimorphic pathogenic mechanisms in GBM [163]. A recent study illustrated in mouse GBM models that M-MDSCs were enriched in the male tumors and PMN-MDSCs were elevated in the blood of females [87]. Proliferating M-MDSCs were associated with poor prognosis of males, and depletion of PMN-MDSCs extended survival only in female mice [87]. These novel findings demonstrate that by multiple mechanisms, MDSC subsets not only modulate the immunosuppressive TME but also drive systemic immune responses in a sex-dependent manner, with implications for personalized therapeutic interventions in GBM.

Up to now, intense research efforts have been focused on developing anti-GBM strategies by blocking MDSC function to remodel the TME [125, 161]. MDSCs can be selectively depleted using 5-flurouracil (5-FU) at a low-dose administration. It was reported that either MDSC depletion by 5-FU or MIF inhibition could result in prolonged survival in syngeneic mouse models of GBM [161, 162]. Although anti-PD-1 monotherapy has failed to show efficacy in randomized phase III clinical trials for either recurrent or newly diagnosed GBM, combined treatments along with targeting of the PD-1/PD-L1 axis are currently under investigation [125]. CCR2 inhibition was reported to reduce CD11b^+^/Ly6C^hi^/PD-L1^+^ MDSC enrichment in established gliomas and enhance PD-1 blockade efficacy in anti-PD-1 resistant gliomas [125]. One study showed that in GBM-bearing mice, depleting MDSCs using the antibody against Gr1, an immune marker for mouse MDSCs, strongly enhanced the efficacy of tumor-targeted gene therapy, and when combined with anti-PD-L1 or CTLA-4 treatment, greatly improved overall animal survival [164]. Preclinically low-dose chemotherapy proved to be effective in reducing MDSCs [165]. In a clinical trial (NCT02669173), metronomic capecitabine combined with bevacizumab (anti-VEGF) treatment demonstrated safety in GBM patients and led to a marked reduction in circulating MDSC levels and an increase in cytotoxic immune infiltration in the TME, including CD8^+^ effector-memory T cells and NK cells [166]. Other clinical trial efforts to reduce MDSCs in patients are underway, including the use of tadalafil during chemoradiation in newly diagnosed high-grade gliomas (NCT04757662). Investigations are actively underway to define the mechanisms of MDSC expansion, trafficking, and activation in GBM. Several immunosuppression mechanisms discovered in other solid tumors are also applicable for GBMs, such as ROS induction. Further investigation directed at MDSC-mediated immunosuppression holds promise for the discovery of potential diagnostic and therapeutic biomarkers as well as potentially improved immunotherapies for GBM patients.

### Neutrophils

Neutrophils are the frontline of the innate immune system and defend against infection. Neutrophils are normally in the circulation and can be recruited into tissues by chemotactic signals, such as IL-8 and C5a. Neutrophils display plasticity under diverse inflammatory contexts, including cancer [167]. In the TME, depending on their functional state, neutrophils can be classified into pro-inflammatory/anti-inflammatory or pro-tumor/anti-tumor neutrophils [167]. In breast cancer, neutrophils have been reported being involved in cancer metastasis through regulation of arachidonate 5-lipoxytenase (Alox5) activity [168]. Neutrophils were also identified to be one of the major immune cell subsets in GBM TME, alongside TAMs and T cells [91, 169]. During recent years, neutrophils are gaining more attention in GBM progression and prognosis.

In GBM patients, neutrophils accumulate systemically and intratumorally, and are negatively associated with patient prognosis [170–172]. Glioma cells can secrete IL-8, MIF, and CXCL8, which induce neutrophil infiltration into the tumor site [161, 173, 174]. Neutrophils are commonly located in the tumor core of GBM and can produce elastase that facilitates tumor cell infiltration [175, 176]. In addition, neutrophil accumulation upregulated S100A4 expression in GBM cells, which promotes GBM invasion and is a critical regulator of GSC self-renewal [177]. Both *in vitro* and *in vivo* tumor xenograft assays show that the expression of CD133, known as a GSC marker, is associated with increased neutrophil recruitment and IL-1β signaling, underlining the crosstalk between GSCs and neutrophils [178]. Furthermore, neutrophils were found to degranulate and release the bulk of arginase to suppress T cell function in GBM patients [179]. Neutrophil infiltration and neutrophil extracellular trap (NET) formation were found to be increased in high-grade gliomas compared to low-grade gliomas [180, 181]. Tumor-infiltrating neutrophils can induce NET formation [182]. NET overproduction can activate the NF-κB pathway which in turn promotes IL-8 secretion in tumor cells and subsequently enlists more neutrophils into the tumor site to increase NETs formation [181]. It has been reported that assembled NETs could strongly accelerate tumor cell expansion and spread [181–184]. Recent evidence showed that certain tumor damage during early tumor progression (i.e., ischemia) may recruit neutrophils to the tumor site and lead to neutrophil-induced ferroptosis to amplify tumor necrosis and induce invasion in GBM progression [185]. In short, the mechanisms of neutrophils contributing to GBM progression are multifactorial, and notably, the mutual adaptation between GSCs and neutrophils induces tumorigenic phenotypes in neutrophils.

Several studies have demonstrated that tumor-infiltrating neutrophils and elevated neutrophil/lymphocyte ratio (NLR) are associated with poor clinical outcome in GBM patients [170, 171, 186–188]. NLR, a marker of the systemic inflammatory response, has been shown to be a poor prognostic factor for survival in GBM [170, 171, 189, 190]. NLR higher than 4 has been associated with a worse prognosis when detected before treatments [170], during radiation/chemotherapy [171], after the second surgery [189], and in recurrent GBM before treatments [190]. Additionally, tumor-infiltrating neutrophils were found to be correlated with acquired resistance to anti-VEGF therapy [186, 191]. Depletion of neutrophils using antibodies against Ly6G prolonged the survival of mice with developing gliomas [192]. The results from a recent clinical trial (NCT01836536) showed that basal neutrophils are predictive for response to bevacizumab in recurrent GBM patients [193]. Altogether, these studies have underlined that the numbers of neutrophils in the circulation and GBM tumors could be harnessed as tumor progression indicators or prognostic markers for GBM patients.

### T cells

T-cell immunity is critical for the anti-tumor immune response. In contrast to B lymphocytes, which eliminate exposed antigens through antibody production, naïve T cells can develop and differentiate into several distinct subpopulations: cytotoxic CD8^+^ T cells, the main force for anti-virus/tumor response; helper CD4^+^ T cells, which can further differentiate into Th1, Th2, or Th17 cells depending on environmental cytokines and perform distinct functions; CD4^+^ Tregs, also known as suppressor T cells, which maintain tolerance to self-antigens and suppress the proliferation and function of effector T cells; and memory T cells (CD45RO^+^), including central (*T*_CM_, CCR7^+^CD62L^+^) and effector (*T*_EM_, CCR7^−^ CD62L^−^) types, which can quickly convert and proliferate into large numbers of effector T cells upon re-exposure to cognate antigens.

**Fig. 4:**
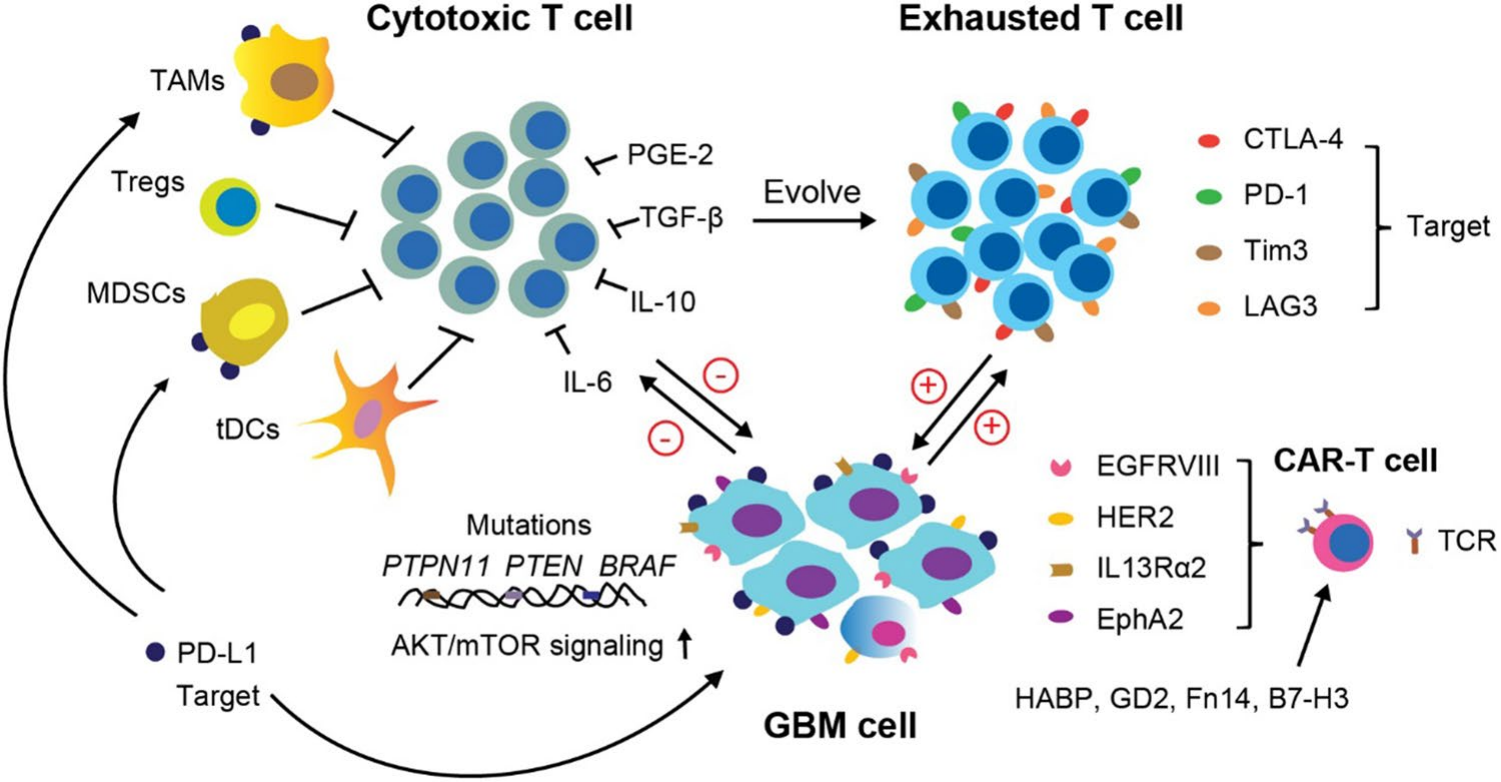
T cell dysfunction in GBM. Immunosuppressive cells (TAMs, Tegs, MDSCs, and tolerogenic DCs) and inhibitory molecules (PGE2, TGF-β, IL-10, and IL-6) induce cytotoxic T cells into the exhausted state. Exhaustion makers (PD-1, CTLA-4, Tim3, and LAG3) are upregulated in dysfunctional T cells. Tumor cells expressing mutant genes (*PTPN11*, *PTEN*, and *BRAF*) exacerbate T cell anergy. The abnormal signaling pathway (AKT/mTOR) activation promotes tumor growth and invasion. CAR-T cells with modified TCRs targeting IL-13Rα2, HER2, EphA2, or EGFRvIII have been tested in clinical trials for GBM. New antigens for CAR-T cells tested in animal models include HABP, GD2, Fn14, and B7-H3. Targets used for immune checkpoint blockade therapies include CTLA-4, PD-1, and PD-L1. Tolerogenic DCs and tDCs

Since most tumor-associated antigens are overexpressed or mutant self-components, it is hard for the immune system to recognize and eliminate early tumor-initiating cells. Thus, CSCs may escape from immune attack and contribute to tumor recurrence. In addition, CSCs can remodel the tumor ecosystem via different molecular and cellular mechanisms to facilitate tumor growth, invasion, and distal spread [194]. In solid tumors, the dysfunctional state of T cells refers to “exhaustion.” Exhausted T cells are featured by weak or no response to antigens; loss of effector function (cytokine secretion and cytotoxicity); upregulation of inhibitory receptors such as PD-1, CTLA-4, Tim3, and LAG3; and metabolic reprogramming [194, 195]. In the TME, signals that lead to T cell exhaustion are from antigens; co-stimulation; T cell inhibitory cells such as Tregs, TAMs, MDSCs, and tolerogenic DCs (tDCs); and metabolic pathways as well as soluble factors [196]. Particularly, once T cells are excessively and continually exposed to low-immunogenic antigens or inflammatory signals, normal function will be disrupted, leading to flawed T cells (Fig. 4) [197].

As mentioned above, T cells occupy only around 10–15 % of leukocytes that are present in GBM TME [86, 91, 95]. Among all CD3^+^ T cell populations, there are few naive T cells, and CD4^+^ and CD8^+^ memory T cells dominate the landscape [86, 91]. Missing naïve T cells are found to be sequestered in large numbers in the bone marrow [198]. Sequestration of T cells in bone marrow is thought to be a tumor-adaptive mode of T cell dysfunction [199]. Moreover, in *IDH1*^wt^ GBM, most T cells are anergic due to constant interaction with an abundance of TAMs [95]. Immune checkpoint blockade including anti-PD-1/PD-L1 and anti-CTLA-4, designed to amplify endogenous anti-tumor T cell responses, has showed much success in certain cancers, such as melanoma and non-small cell lung cancer [200, 201]. However, anti-PD-1 monotherapy has failed to show efficacy in randomized phase III clinical trials for either recurrent (NCT02017717) or newly diagnosed GBM (NCT02617589) [200, 202, 203]. Furthermore, earlier studies have shown that tumors with greater mutational loads, expressing more tumor-specific antigens that induce larger amplitude of neoantigen-specific CD8^+^ T cell responses, are more sensitive to anti-PD-1 therapy, such as non-small cell lung cancer [204]. However, the absence of many neoantigens in GBM cells makes it challenging to achieve durable and effective anti-tumor immunity.

Moreover, immunosuppressive cells, such as TAMs, MDSCs, and Tregs that maintain an immunoinhibitory TME and inhibit T cell functions, are observed to be increased in GBM patients [86]. As in other tumor types, a pro-tumoral cytokine network induced by CD4^+^ T and myeloid-lineage cells in the TME, including prostaglandin E2 (PGE-2), IL-6, IL-10, and TGF-β, exacerbates the repression of effector CD8^+^ T cell response and induces Tregs [205]. A subset of IL-10-releasing HMOX1^+^ myeloid cells, spatially located around mesenchymal-like tumor cells, was identified to drive T cell exhaustion, and contribute to the immunosuppressive TME [206]. Genomic and expression-profile analysis revealed that specific molecular mutations in GBM are associated with immunosuppressive signature expression, such as *PTEN*, *PTPN11*, and *BRAF*, which further complicates immunotherapy treatment options [207]. Thus, the compromised cytotoxicity of CD8^+^ T cells together with the heterogeneity of tumor cell intrinsic mutations are major obstacles to improve the therapeutic efficacy of checkpoint inhibitors in GBM. Furthermore, there is evidence that enhanced inhibitory-receptor signals in T cells, such as CTLA-4 and PD-1, restrain glucose consumption and glycolytic capacity, leading to dampened mTOR activity and IFN-γ production, thereby allowing tumor progression [208, 209]. Oppositely, high levels of PD-L1 expression in tumor cells can activate the Akt/mTOR pathway and strengthen glycolytic process [209]. This rewired glucose metabolism restricts effector T cell function and favors GBM cell survival and tumor progression.

Although at present the clinical response of checkpoint blockade therapy in GBM is not ideal, studies to improve the therapeutic efficacy are in progress. There are several approaches for intensified therapeutics: (1) multi-target treatment regimens, incorporating treatments that target intracellular inhibitory signaling activities of immune cells or metabolic pathways of tumor cells; (2) tumor TME remodeling, for instance, cytokine therapy to attract more T cells and reduce the accumulation of suppressive cell subsets; (3) synthetic modification of T cell, to achieve long-lived or enhanced effector function. Lately, a combination of anti-VEGFR2 and anti-PD-L1 treatment was unsuccessful in achieving better therapeutic efficacy in GBM [209]. Subsequent studies indicated that increasing the intratumoral formation of high endothelial venules (HEVs) could sensitize tumors to antiangiogenic/anti-PD-L1 therapy, which was associated with reinforced lymphocyte infiltration and activity [210]. Notably, more investigations have focused on T cell engineering that aims at potentiating antigen recognition specificity and effector function via genetic TCR reprogramming [211]. TCR α and β chains are rearranged via molecular genetic enzyme-tools and generate so-called chimeric antigen receptor-T cells (CAR-T cells) [212]. To date, CD19-targeted CAR-T therapy has exhibited unprecedented success in the treatment of B cell malignancies [212, 213]. However, a major concern for widespread use of CAR-T therapy in cancers lies in potential severe side-effects, such as “cytokine storm” and neurotoxicity. For solid tumors, cytotoxicity is not limited to tumor cells and can damage healthy vital tissues [212]. How to mitigate the off-tumor effects and nerve injuries is being carefully investigated. It is noteworthy that recent clinical trials have shed light on the feasibility and safety of CAR-T cell therapy for GBM [214]. Three different antigen-targets are under investigation for GBM including IL13Rα2, HER2, and EGFRvIII (a mutant isoform with deletion at exons (2–7)), based on the expression specificity and levels in tumor cells [215]. In an early clinical trial (NCT02208362), IL13Rα2-directed CAR-T cells were delivered into the cerebrospinal fluid, and one remarkable patient experienced tumor regression, even though subsequent recurrence emerged [216, 217]. Promisingly, no toxicity beyond grade 3 was observed in this trial [216, 217]. At present, IL13Rα2-targeted CAR-T therapy combined with ICB treatment (NCT04003649) is being tested in patients with recurrent GBM [218]. To enhance safety, the TCR of HER2-specific CAR-T cells was further modified with an FRP5-based exodomain and a CD28 signaling endodomain, and CAR-T cells were delivered through peripheral blood infusion in a phase I clinical trial (NCT01109095) [219]. No dose-dependent toxicity was observed, but HER2-specific CAR-T cells did not show expansion in patient blood after infusion, which reduced their effectiveness [219]. A single peripheral infusion of EGFRvIII CAR-T cells was tested in 10 patients with recurrent GBM in a clinical trial (NCT01454596) [220]. EGFRvIII CAR-T cells exhibited obvious intratumoral trafficking and expansion. However, subsequent characterization of the TME revealed that CAR-T cells targeting EGFRvIII triggered a compensatory immunosuppressive response, including increased infiltration of Tregs, upregulated expression of PD-L1, and production of immunoinhibitory cytokines, such as TGF-β and IL-10 [220]. Recently, epitopes of ephrin type-A receptor 2 (EphA2) have been used to generate third-generation CAR-T cells, and anti-GBM efficacy in preclinical models has been tested [221]. The survival of tumor-bearing mice after EphA2 CAR-T cell treatment was significantly improved [221]. The first-in-human trial of EphA2-directed CAR-T cells in patients with recurrent GBM demonstrated that EphA2 CAR-T cells were preliminary tolerable with at least transient clinical efficacy [222]. In addition, several new antigen-targets for CAR-T cells have been tested in animal models of GBM, such as HABP, GD2, Fn14, and B7-H3 [223–227]. Notably, a single intravenous infusion of GD2 CAR-T cells following radiation treatment led to complete antitumor response in advanced syngeneic orthotopic GBM models [224]. It seems in these early studies CAR-T cells have demonstrated safety and some effectiveness for GBM immunotherapy and could be further pursued in a combinatorial therapeutic modality.

### NK cells

Natural killer cells, known as NK cells among innate immune cells, play analogous roles as cytotoxic CD8^+^ T cells and are another central cell population for anti-tumor immunity. NK cells can be activated by downregulated MHC-I molecule expression on tumor cells and exert direct killing effects on target cells in an antigen-independent manner that echoes and restricts tumor escape from antigen-dependent cytotoxic T cell-mediated effector functions. NK cells can also respond to tumor cell-derived growth factor and secrete cytotoxic cytokines, such as IFN-γ and TNF-α, as well as proinflammatory chemokines to induce tumor cell growth arrest [228]. Additionally, the direct interaction between NK and tumor cells can downregulate tumor cell-cycle genes and inhibit cell proliferation [228]. However, to date, extensive studies into the TME landscape have revealed that NK cells are heterogeneous and can be defined into two subsets: immature CD56^int/bright^CD16^−^ cells, and fully mature CD56^int^CD16^+^ cells with maximum cytotoxic capacity [229]. In GBM tumors, the frequency of NK cells among the infiltrative lymphocyte populations is approximate 20%, and the immature CD56^int/bright^ CD16^−^ NK cells predominate [86]. NK cell proportions are not significantly increased at the tumor site compared with that in the peripheral blood of GBM patients [230]. Moreover, NK cells that are recruited into the tumor site express high levels of CXCR3, and are no longer cytolytic, as these immature NK cells express low levels of IFN-γ [231]. The limited functional capacity of immature NK cells contributes to TME immunosuppression and may promote GBM malignant progression.

In the last 20 years, multiple groups have investigated the role of NK cells, with the purpose of creating novel therapeutic approaches for GBM [232, 233]. NK cells express both activating and inhibitory receptors on the cell surface. The interaction between these receptors and corresponding ligands on the target cell surface determines the action of these lymphocytes [234]. A few studies have concentrated on utilizing oncolytic herpes simplex virus to achieve GBM virotherapy [235, 236]. In these studies, to reduce viral clearance and enhance the efficacy of viral oncolysis, mouse GBM cells were engineered to express a ligand for KLRG1, an inhibitory receptor expressed on NK cells [235, 236]. However, the improved survival in GBM-bearing mice was found to be related with increased intratumoral viral spread rather than the inhibition of NK cell activity [236]. As insights into the TME of GBM deepen, more work has been focusing on promoting NK cell maturation and intensifying their activity in GBM immunotherapy [237–242]. One initial clinical trial collected peripheral blood mononuclear cells (PBMCs) from patients with recurrent GBM and performed autologous NK cells ex vivo expansion [237]. NK cell re-infusion was found to be safe and partially effective in suppressing tumor progression [237]. Based on these results, an improved regimen was subsequently posed–that NK cell treatment should be combined with IL-2 infusion or radiation therapy [237]. In addition, some chemical inhibitors could sensitize tumor cells for NK cell-mediated cytotoxicity and were suggested to be applied together with NK cell treatment, such as the histone deacetylase inhibitor trichostatin A and proteasome inhibitor bortezomib [238, 239]. *In vivo* experiments showed that these inhibitors could enhance NK cells-mediated tumor recognition and lysis [238, 239]. Additionally, in a rat GBM model, investigators tested the combined treatment of NK cells and antibody-mediated anti-angiogenesis, resulting in prolonged survival and reduced tumor size [240]. KIR2DS2 has been identified as a potent activating receptor on the NK cell surface, and its effect is independent of other activating or inhibitory receptors [241]. One study found that human allogeneic NK cells with the KIR2DS2 immunogenotype exhibited stronger cytotoxicity towards patient-derived GBM cells both *in vitro* and *in vivo* [242].

Recently, NK cells have been modified with CARs, endowing these cells with tumor recognition specificity and augmenting tumor killing effects [243]. Similar to CAR-T cells, therapeutic targets designed in CAR-NK cells for GBM treatment include EGFRvIII, EGFR, and HER2, which have been investigated in several preclinical studies [243]. One study generated an EGFRvIII-specific NK cell line by reforming human NK-92 cells that possess typical phenotypes of activated NK cells, to express the binding-domain of EGFRvIII-specific antibody and signaling domains of CD28 and CD3ζ [244]. This CAR-engineered NK-92 cells slowed the growth of EGFRvIII-positive GBM xenografts and improved survival in an immunodeficient mice model [244]. Given that heterogeneous tumors express both EGFR and EGFRvIII, and single-target treatment has resulted in tumor tolerance and recurrence in some GBM patients, dual-specific second-generation CAR-NK cells were created and led to a more pronounced progression-free survival in EGFR and EGFRvIII double-positive mouse tumor models [244]. A recent study transduced human NK cell line KHYG-1 with lentiviral vectors expressing EGFRvIII-specific scFv, and the EGFRvIII specific-CAR-KHYG-1 cells inhibited GBM cell growth by induction of cell apoptosis [245]. Additionally, HER2-specific NK-92 cells displayed high and selective cytotoxicity against HER2-positive target cells in established orthotopic GBM xenograft models [246, 247]. The phase I clinical trial CAR2BRAIN (NCT03383978) evaluated the safety and tolerability of HER2/ErbB2-specific NK-92/5.28.z cells, one clone of modified HER2-specific NK-92 cells [243]. Cells were injected into the wall of the resection cavity during relapse surgery in GBM patients, and no dose-limiting toxicities have been observed [243]. These preclinical and clinical data demonstrate that CAR-NK cells can be further developed for clinical application. In addition, CD155, recently identified as a pro-tumorigenic gene, is overexpressed on GBM tumor cells and modulates GBM invasiveness and progression [248]. One of the mechanisms posited is that CD155 weakens NK cell function by interaction with the inhibitory receptor TIGIT [248], suggesting CD155 could be a potential target for NK cell-based immunotherapy. GSCs, a source of GBM growth and recurrence, have been shown to be more susceptible to NK cell-mediated lysis than differentiated tumor cells [249], which implies that NK cell therapy might be more important than cytotoxic T cells in GSC eradication. Overall, immunotherapy with NK cells seems to be a promising strategy for treating GBM patients, and further investigation is needed.

## Glioma stem cells

A number of studies in GBM using both transgenic models and human specimens have verified the existence of a malignant, neural progenitor-like population in GBM, which has been called “glioma stem cells” (GSCs) or brain tumor-initiating cells, and is thought to drive therapy resistance and recurrence [250–254]. The functional definition of GSCs is based on three biological properties: self-renewal and multipotency, which are properties of normal stem cell populations, and, most importantly, *in vivo* tumorigenicity [255]. A recent study mapped GBM tumor cells to the cellular lineage hierarchy of the developing human brain by single-cell RNA sequencing on adult GBM cells and normal human fetal brain cells. These analyses identified three conserved neurodevelopmental lineages in GBM and a glial-like progenitor population that contains the majority of mitotic cells and drives tumor growth and chemoresistance [256]. GSCs were found to be more resistant to radiotherapy and TMZ chemotherapy, consistent with their role in relapse after treatment [252, 253]. Accordingly, transcriptional signatures of GSCs were clinically associated with patient outcome [257, 258]. Wnt signaling and the PI3K/Akt/mTOR pathway have been intimately linked with GSC chemotherapy resistance as well as proliferation, metabolism, and survival [259]. Overall, there is abundant evidence for the critical role of GSCs in GBM initiation, growth, and treatment resistance.

**Fig. 5:**
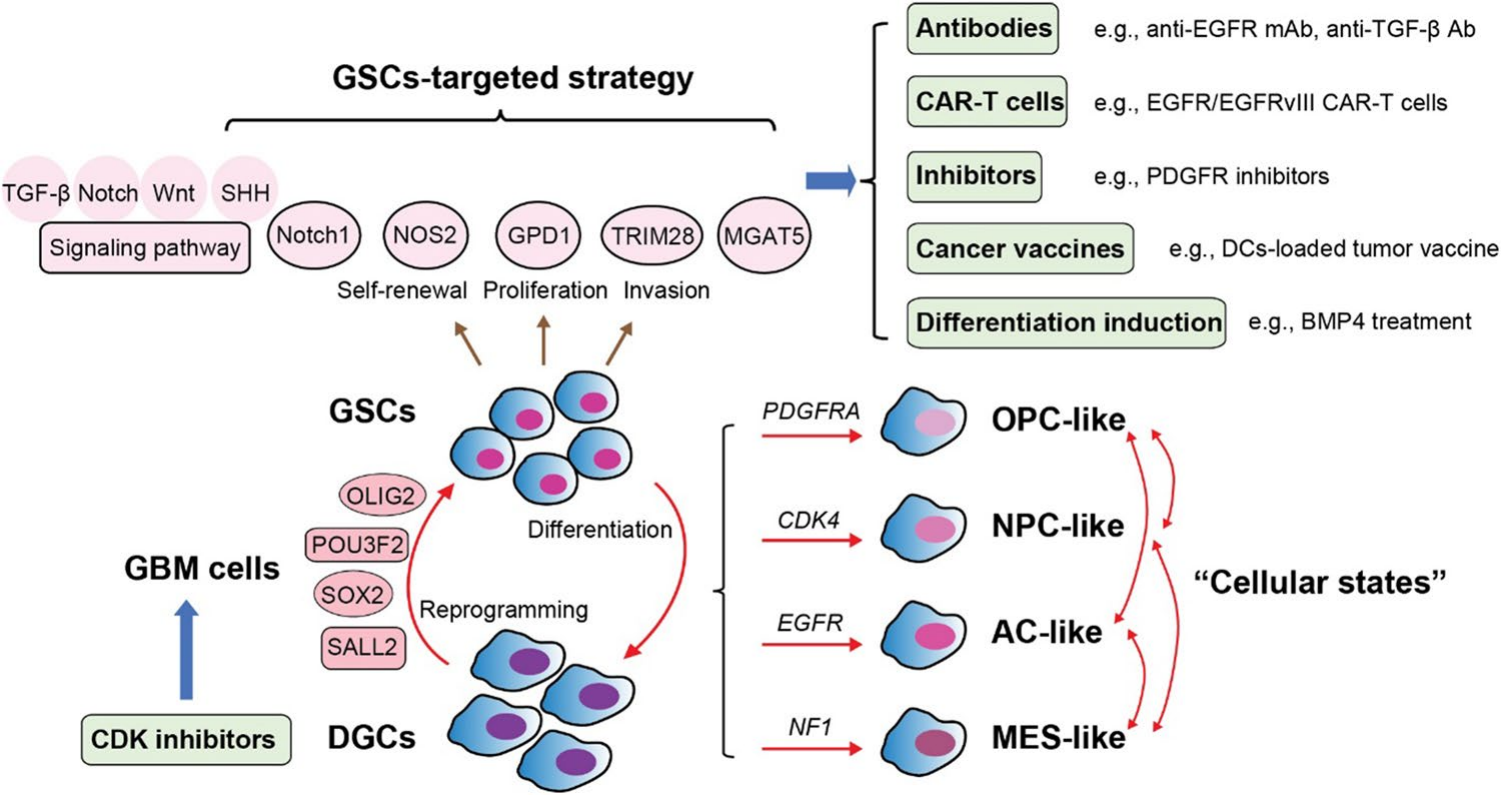
Plasticity in GSC phenotype and relevant therapeutic strategies. Four core transcription factors, POU3F2, SALL2, SOX2, and OLIG2 are sufficient to reprogram differentiated glioma cells (DGCs) into GSCs. There are four major cellular states of GBM cells including neural progenitor-like (NPC-like), oligodendrocyte-progenitor-like (OPC-like), astrocyte-like (AC-like), and mesenchymal like (MES-like) states within one GBM sample. The relative frequencies of each state are associated with genetic alterations in *CDK4*, *PDGFRA*, *EGFR*, and *NF1* that each mutation favors a state. Based on the feature of GSCs, several therapeutic approaches are being evaluated including antibodies (Abs), CAR-T cells, inhibitors, cancer vaccines, and induction of GSC differentiation. CDK inhibitors that lead to cell growth arrest can be incorporated into GSC-based therapy regimens

Numerous studies to date have revealed that the GSC state is epigenetically driven and is highly dynamic and plastic (Fig. 5). In an important earlier study, Suva and colleagues identified that forced expression of four core transcription factors, POU3F2, SALL2, SOX2, and OLIG2, is sufficient to reprogram differentiated GBM cells into tumor-propagating GSCs [260]. An overall single cell survey of the heterogeneous cell states in GBM was performed by Neftel et al., who found that malignant cells in GBM can be classified into four major cellular states including neural progenitor-like (NPC-like), oligodendrocyte-progenitor-like (OPC-like), astrocyte-like (AC-like), and mesenchymal like (MES-like) states, all of which exhibit a subset of cells with proliferative capacity, thus potentially expanding the phenotype of GSCs beyond a simple binary (i.e., stem/non-stem cell) state [261]. Each GBM sample contains cells in multiple states and the relative frequency of each state varies between tumors. Interestingly, the frequencies of each of the four cellular states were associated with genetic alterations in *CDK4*, *PDGFRA*, *EGFR*, and *NF1*, with each gene mutation favoring a particular state [261]. The interplay between the epigenetic states and potential genetic drivers appears to create a spectrum of heterogeneous GSCs, which provide the driving force for tumor growth and maintenance [262–265]. Using two human neural stem cell (NSC)-derived GBM models, one group of researcher demonstrated GBM progression is primarily driven by multi-step transcriptional reprogramming and fate-switches in the NSC-like cells, which sequentially generated heterogeneity and induced phenotype transitions [266]. Additionally, studies of inter-tumor heterogeneity suggest that at least three subtypes of GBM tumors exist, namely, proneural (TCGA-PN), classical (TCGA-CL), and mesenchymal (TCGA-MES) [267]. Tumoral multi-region analysis has shown that diverse subtypes can co-exist in different regions of the same tumor, and subtypes can change over time and through therapy [254, 268].

Research around GSCs is critical to understand the mechanisms of GBM invasiveness and migration (Fig. 5). CD133^+^Notch1^+^ GSCs were shown to be preferentially located along white matter tracts, at the invasive front of GBMs [269]. A positive-feedback circuit between nerve fibers expressing the Notch ligand Jagged1 and GSCs was found to enhance Notch1-SOX2 signaling in GSCs and promote GSCs invasion along white matter tracts [269]. It was also reported that Notch1 modulated the self-renewal and invasion of GSCs via induction of CXCR4 in GSCs [270]. GPD1 was found to be specifically expressed in GSCs and not in normal NSCs [270]. GPD1^+^ GSCs were enriched at tumor borders and shown to drive tumor relapse after chemotherapy [271]. Compared to non-GSCs and normal progenitors, GSCs harbor increased oxide synthase-2 (NOS2) activity which induces genes regulating tumor growth and distal spread [272]. GSCs exhibit higher expression of TRIM28, a biomarker for GBM, and anti-TRIM28 treatment inhibited GBM cell invasion and spread both *in vitro* and *in vivo* [273]. *HOXA5* gene amplification is an independent prognostic factor for worse outcomes in GBM patients [274]. HOXA5 can activate *PTPRZ1* transcription and PTPRZ1-initiated signaling, which increased proportions of GSCs and promoted the self-renewal and invasiveness of these cells [162]. MGAT5 can regulate GSC invasion by catalyzing multibranched N-glycans to increase GSC mechanotransduction, thereby promoting GSCs spread [275]. Moreover, using an immortalized human NSC-derived GBM model, one study found that intrinsic PAX6/DLX5 transcriptional regulation in GSCs drove WNT5A-mediated transformation of GSCs into endothelial-like cells (GdECs), and GdECs could recruit existing endothelial cells to build an invasive front, contributing to infiltrative growth, distant migration, and recurrence [276]. GSCs have also been shown to recruit monocytes to the TME and polarize them to a pro-tumor phenotype via secretion of CCL2 and CSF-1 [277]. Finally, GSCs can directly inhibit effector T cell proliferation and function and induce T cell apoptosis [278]. Thus, GSCs contribute to maintaining immunosuppression within the TME.

Ongoing work and current clinical trials have been developed to therapeutically target GSCs directly or indirectly by altering the GSC-supportive TME. Several therapeutics are being evaluated including monoclonal antibodies (mAbs), GSC-associated antigen-based CAR-T cells, inhibitors, cancer vaccines, and induction of GSC differentiation (Fig. 5) [279]. Tumor-associated antigens (TAAs) are proteins that are significantly over-expressed in cancer cells compared to normal tissue. Peptides of these TAAs, presented by MHC molecules, can be recognized by antibodies and T cells that initiate anti-tumor immune responses. Amplification and mutation of *EGFR* represent common genetic signatures in GSCs, and mAbs directly targeting EGFR have been utilized as a well-known therapeutic approach in GBM. For instance, anti-EGFR mAbs, such as Cetuximab, panitumumab, and nimotuzumab, can functionally prevent EGFR-mediated signaling and decrease GSC proliferation [280–282]. The efficacy of EGFR/EGFRvIII-targeted CAR-T cells in inhibiting GSC function is being evaluated in both preclinical and clinical studies. Similar to EGFR, HER2-specific CAR-T cells are under investigation for its role in anti-GBM therapy. PDGFR family members are commonly overexpressed and/or mutated in proneural-subtype GBMs and contribute to the self-renewal and tumor-initiating capacity of GSCs [283, 284]. Tandutinib, a PDGFR inhibitor, was demonstrated to inhibit GBM growth in animal models but did not show significant therapeutic effects in patients with recurrent GBM [285]. Several signaling pathways were reported to be highly active in GSCs and can be targeted, such as TGF-β, Notch, Wnt, and SHH [269, 270, 276, 286–289]. GC1008, the anti-TGF-β neutralizing antibody, inhibited tumor cell invasion into adjacent areas of the brain in a GL261 GBM model [290]. However, no clinical benefit of GC1008 was observed in a phase II clinical trial (NCT01472731) [290]. Targeting the Notch pathway has been carried out through therapeutic downregulation of key molecules in the signal transduction cascade, such as Notch, Delta-like-1, Jagged-1, γ-secretase, ADAM10, and ADAM17 [291]. Notably, the γ-secretase inhibitor RO4929097 is being tested in several clinical trials [291]. Although the Wnt signaling pathway is pivotal in modulating the differentiation status and proliferation of GSCs, inhibition of the pathway causes serious side effects given that a considerable number of physiological processes depend on it [292]. The SHH signaling pathway is involved in the self-renewal of GSCs and also contributes to chemo-resistance to TMZ [293]. Studies targeting the SHH pathway alone or in combination with other therapeutic approaches are being performed [294, 295].

Distinct from mAb and CAR-T cell therapies, cancer vaccines belong to a class of active immunotherapies which mobilize the host immune system to recognize tumor components in advance and kill tumor cells once they emerge. Components from GSCs can be utilized to develop cancer vaccines, with the aim to eradicate tumor growth and prevent recurrence. In the first GSC-targeted vaccine therapy in humans (NCT00846456), patients-derived GSCs were expanded *in vitro*, and the isolated mRNA was transfected into monocyte-derived DCs. This DC-loaded tumor vaccine was well-tolerated in GBM patients without causing serious adverse reactions [296]. A recombinant TAA peptide was made from epitopes derived from several TAAs overexpressed in GSCs, including HER2, AIM-2, gp100, IL13Rα2, TRP-2, and MAGE1 [297]. DCs pulsed with this peptide, namely ICT-107 have been used in a phase II (NCT01280552) clinical trial recently [145]. Although ICT-107-treated GBM patients showed improved PFS, there were concerns about the clinical relevance of ICT-107, since only HLA-A1 positive, but not HLA-A2 positive, vaccinated patients displayed a significant OS benefit [298]. Furthermore, SOX2 has been found to be enriched in the GSC population and play a critical role in the maintenance and chemoresistance of GSCs [299]. SOX2-targeted vaccines may represent a new direction in the future.

GSCs can become more sensitive to therapy after differentiation into more terminal GBM cells. Hence, several studies have tried the approach of inducing GSCs differentiation for anti-GBM treatment. For instance, bone morphogenic proteins (BMPs) can induce GSC differentiation. In one study, BMP4 was shown to downregulate the levels of SOX2 and OLIG2 in GSCs, and induce the expression of GFAP [300]. BMP4-treated EGFR^high^ GSCs were more sensitive to TMZ *in vitro* [300]. Recently, the overexpression of miR-128 or miR-302a has been shown to promote GSC differentiation and enhance senescence mediated by axitinib treatment, a tyrosine kinase inhibitor [301]. In addition, in view of the trophism of NSCs into tumor GSCs niche, many stem cell-based therapeutics have been tested, including carrier strategies to deliver viral particles, prodrugs and cytokines [302]. However, concerns regarding the safety and ethics of stem cell therapy may need to be addressed for clinical translation. In summary, intratumoral GSC removal will be essential for a curative approach for GBM.

## CDK inhibitors

An important feature of GBM is the very high rate of mitosis. Cell proliferation dysregulation in cancer is often mediated by alterations in cyclin-dependent kinase (CDK) activity [303]. CDKs are a family of conserved serine/threonine protein kinases, and certain members play an essential role in cell cycle regulation to ensure homeostasis and maintenance of normal cell proliferation [304]. The deregulation of CDK activity in cancers stems from genetic or epigenetic changes in either CDKs, their regulators, or upstream mitogenic pathways. Aberrant CDK activity leads to sustained proliferative signaling and promotes malignant transformations. Similar to GSC-based treatments, targeting abnormal cell cycles may undermine rapid tumor expansion. Thus, therapeutic strategies that block CDK activity can be incorporated with other treatment modalities such as chemotherapy, radiation, GSC-based therapy, or ICB treatment (Fig. 5). Among human CDKs, CDK2 has been found to be significantly enriched in GBM tumors, and CDK2 inhibition reduced GBM cell proliferation and invasion and increased sensitivity to radiation both *in vitro* and *in vivo* [305, 306]. Moreover, CDK1 can phosphorylate the SUMO-specific enzyme, UBC9, which mediates CDK6 SUMOylation during mitosis, and SUMOylated CDK6 drives the cell cycle through G1/S phase transition [307]. Flavopiridol, one of the first generation CDK inhibitors, has been tested in a mouse xenograft model of GBM, though it exhibits a broad spectrum of inhibitory activity against CDKs [308]. Flavopiridol showed promising results in combination with TMZ, as it enhanced cytotoxicity in GBM cells and sensitized xenografted mice to TMZ [308]. Subsequently, to increase the selectivity of CDK inhibitors towards CDK1 and CDK2, several compounds were developed as second-generation CDK inhibitors such as roscovitine, PHA-848125, dinaciclib, and Purvalanol A [309]. Preclinical investigations have revealed that these inhibitors exhibited significant cytotoxicity against GBM [309]. However, none of them has moved into phase I clinical trials. In addition, the cyclin D1-CDK4/6-Rb1 pathway is altered in nearly 80% of human gliomas. Two highly selective CDK4/6 inhibitors, palbociclib and abemaciclib, significantly increased the survival in a rat orthotopic U87MG xenograft model compared with vehicle-treated animals [309]. Of note, Abemaciclib is being evaluated in phase I clinical trials for several solid cancers, including GBM [310]. Recently, ribociclib, a CDK4/6 inhibitor, has been tested in patients with recurrent GBM in a phase Ib study (NCT02345824) [311]. Although no serious adverse events were observed, ribociclib monotherapy showed no clear clinical benefit for the treatment of recurrent GBM [311]. In the future, advances in stratifying patient populations and in CDK drug design may offer new hope for this therapeutic direction. In addition, combinatorial therapeutics incorporating CDK drugs is being investigated currently [312].

## Conclusion

The utilization of various high-throughput sequencing technologies has recently expanded our understanding of the heterogeneity of GBM TME, and exploration of individual components has uncovered important crosstalk between tumor cells and their microenvironment. Both tumor-intrinsic and extrinsic heterogeneity contribute to the complexity of the TME landscape [313]. Generally, tumor cells, and in particular GSCs, form a unique neural ecosystem and trigger a dynamic evolutionary process that reorganizes the TME, generating an immunosuppressive niche that reprograms both local and recruited immune cells to form a tumor permissive status. Therefore, to understand the mechanisms modulating GBM progression and recurrence, an exhaustive inquiry into the role of each TME component is indispensable, including ECM composition, diverse immune cell populations, and heterogeneous GSCs. So far, the exploration of individual components in the TME has identified multiple potential therapeutic targets, some of which have been evaluated in both preclinical and clinical investigations. Emerging new sequencing technology is able to integrate transcriptional profiles and intracellular metabolic activities of multiple cell types in GBM tumors, which will be valuable for defining new treatment targets [314]. In addition, different treatment modalities are being tested, for instance, nano-drugs and laser interstitial thermal ablation [315–317]. Further studies on combinatorial therapy regimens are expected to improve patient overall survival in the near future.

## Abbreviations

AC: Astrocyte
ADAM: A disintegrin and metalloproteinase domain-containing protein
AIM-2: Absent in melanoma 2
Akt: Protein kinase B
ALT: Alanine aminotransferase
Alox5: Arachidonate 5-lipoxytenase
AST: Aspartate aminotransferase
BBB: Blood-brain barrier
Bcl-2: B cell lymphoma 2
Bcl-xL: B cell lymphoma extra-large
BMPs: Bone morphogenic proteins
CAR: Himeric antigen receptor
CCL2: C-C motif chemokine ligand 2
CCR2: C-C motif chemokine receptor 2
CDK: Cyclin-dependent kinase
CK2: Casein kinase 2
CNS: Central nervous system
CSCs: Cancer stem cells
CSF1: Colony-stimulating factor 1
CSPG: Chondroitin sulfate proteoglycan
CTLA-4: Cytotoxic T-lymphocyte-associated protein 4
CXCL8: C-X-C motif chemokine ligand 8
CXCR3: C-X-C motif chemokine receptor 3
DCs: Dendritic cells
DLX5: Distal-less homeobox 5
ECM: Extracellular matrix
EGFRvIII: Epidermal growth factor receptor variant III
EGF: Epidermal growth factor
EMT: Epithelial-to-mesenchymal transition
EPHA2: Ephrin Type-A Receptor 2
FN: Fibronectin
Fn14: Fibroblast growth factor-inducible 14
GAGs: Glycosaminoglycans
GBM: Glioblastoma
GdECs: Endothelial-like cells
GFAP: Glial fibrillary acidic protein
GM-CSF: Granulocyte-macrophage colony-stimulating factor
GPD1: Glycerol-3-phosphate dehydrogenase 1
GSCs: Glioma stem cells
GWAS: Genome-wide association study
HA: Hyaluronic acid
HABP: Hyaluronan binding protein
HER2: Human epidermal growth factor receptor 2
HEVs: High endothelial venules
HIF1A: Hypoxia-inducible factor 1-alpha
HMOX1: Heme Oxygenase 1
HOXA5: Homeobox protein Hox-A5
ICAM-1: Intercellular adhesion molecule 1
ICB: Immune checkpoint blockade
IDH: Isocitrate dehydrogenase
IDO: Indoleamine-pyrrole 2,3-dioxygenase
IFN-γ: Interferon‐gamma
KIR2DS2: Killer-cell immunoglobulin-like receptor two domains short tail 2 protein
KLRG1: Killer cell lectin like receptor G1
LOX: Lysyl oxidase
mAbs: Monoclonal antibodies
MAGE1: Melanoma-associated antigen 1
MBVs: Matrix-bound nanovesicles
M-CSF: Macrophage colony-stimulating factor
MDMs: Monocyte-derived macrophages
MDSCs: Myeloid-derived suppressor cells
MES: Mesenchymal
MGAT5: Alpha-1,6-mannosylglycoprotein 6-beta-N-acetylglucosaminyltransferase A
MHC: Major histocompatibility complex
MHC-I: Major histocompatibility complex class I molecules
MIF: Macrophage migration inhibitory factor
miRNA: MicroRNAs
MMP: Matrix metalloprotease
mTOR: Mammalian target of rapamycin
NETs: Neutrophil extracellular traps
NF-κB: Nuclear factor kappa B
NFE2L2: Nuclear factor erythroid 2-related factor 2
NK: Natural killer
NLR: Neutrophil/lymphocyte ratio
NOS2: Oxide synthase-2
NPC: Neural progenitor
NSCs: Neural stem cells
OLIG2: Oligodendrocyte transcription factor 2
OPC: Oligodendrocyte progenitor
OS: Overall survival
PAX6: Paired Box 6
PBMCs: Peripheral blood mononuclear cells
PDGF: Platelet-derived growth factor
PDGFR: Platelet-derived growth factor receptor
PD-L1: Programmed cell death ligand 1
PDTX: Patient-derived glioblastoma xenograft
PD-1: Programmed cell death protein 1
PEG: Polyethylene glycol
PFS: Progression free survival
PGE-2: Prostaglandin E2
PI3K: Phosphatidylinositol 3-kinase
PRRs: Pattern-recognition receptors
PTPRZ1: Protein tyrosine phosphatase receptor type Z1
PYK2: Protein tyrosine kinase 2
Rb1: Retinoblastoma protein
ROCK: Rho-associated coiled-coil kinase
ROS: Reactive oxygen species
scFv: Single chain variable fragment
SHH: Sonic hedgehog
shRNA: Short-hairpin RNA
STAT3: Signal transducer and activator of transcription 3
SUMO: Small ubiquitin-like modifier
TAAs: Tumor-associated antigens
TAMs: Tumor-associated macrophages
TCR: T cell receptor
TGF-β: Transforming growth factor beta
TIGIT: T cell immunoreceptor with Ig and ITIM domains
TLR2: Toll-like receptor 2
TME: Tumor microenvironment
TMZ: Temozolomide
TNF-α: Tumor necrosis factor-alpha
Tregs: Regulatory T cells
TRIM28: Tripartite motif-containing 28
TRP-2: Tyrosine-related protein 2
UBC9: Ubiquitin-conjugating enzyme 9
VCAM-1: Vascular cell adhesion protein 1
VEGF: Vascular endothelial growth factor
VEGFA: Vascular endothelial growth factor A
VEGFR2: Vascular endothelial growth factor receptor 2
WNT5A: Wnt family member 5A
YAP1: Yes-associated protein 1
3D: Three-dimensional
